# Localisation of laminin within *Plasmodium berghei *oocysts and the midgut epithelial cells of *Anopheles stephensi*

**DOI:** 10.1186/1756-3305-1-33

**Published:** 2008-09-22

**Authors:** Adéla Nacer, Karen Walker, Hilary Hurd

**Affiliations:** 1 Centre for Applied Entomology and Parasitology, Institute for Science and Technology in Medicine, School of Life Sciences, Keele University, Keele, Staffordshire, ST5 5BG, UK; 2 Central Electron Microscope Unit, School of Life Sciences, Keele University, Staffordshire, UK

## Abstract

**Background:**

Oocysts of the malaria parasite form and develop in close proximity to the mosquito midgut basal lamina and it has been proposed that components of this structure play a crucial role in the development and maturation of oocysts that produce infective sporozoites. It is further suggested that oocysts incorporate basal lamina proteins into their capsule and that this provides them with a means to evade recognition by the mosquito's immune system. The site of production of basal lamina proteins in insects is controversial and it is still unclear whether haemocytes or midgut epithelial cells are the main source of components of the mosquito midgut basal lamina. Of the multiple molecules that compose the basal lamina, laminin is known to interact with a number of *Plasmodium *proteins. In this study, the localisation of mosquito laminin within the capsule and cytoplasm of *Plasmodium berghei *oocysts and in the midgut epithelial cells of *Anopheles stephensi *was investigated.

**Results:**

An ultrastructural examination of midgut sections from infected and uninfected *An. stephensi *was performed. Post-embedded immunogold labelling demonstrated the presence of laminin within the mosquito basal lamina. Laminin was also detected on the outer surface of the oocyst capsule, incorporated within the capsule and associated with sporozoites forming within the oocysts. Laminin was also found within cells of the midgut epithelium, providing support for the hypothesis that these cells contribute towards the formation of the midgut basal lamina.

**Conclusion:**

We suggest that ookinetes may become coated in laminin as they pass through the midgut epithelium. Thereafter, laminin secreted by midgut epithelial cells and/or haemocytes, binds to the outer surface of the oocyst capsule and that some passes through and is incorporated into the developing oocysts. The localisation of laminin on sporozoites was unexpected and the importance of this observation is less clear.

## Background

Malaria, a vector borne disease caused by an intracellular obligate protozoan parasite of the genus *Plasmodium*, is responsible for the loss of approximately 2 million lives each year [1]. Control of the disease by drug use or elimination of the vector is becoming more difficult as drug resistance in the parasite and insecticide resistance in the mosquito is widespread. Recent efforts have focused on finding new ways to interrupt the transmission of the parasite via mosquitoes; a strategy referred to as transmission blocking. In addition to the use of insecticide treated bednets [2,3] several new approaches are being explored including transmission blocking vaccine development. The current emphasis on the development of new transmission blocking strategies to control malaria, and in particular the genetic manipulation of mosquitoes, make it essential to achieve a better understanding of the interactions between the vector and parasite.

Infection of the mosquito host occurs when *Plasmodium *gametocytes are ingested during a blood meal. Gametogenesis is triggered, allowing the release of the macrogametes from their host red blood cells and the assembly and release of the microgametes; a process termed exflagellation. Fertilisation rapidly follows and a zygote is produced [4]. Once this occurs, the parasite begins to change to become characteristic of the apicomplexan invasive stages. Within 10–25 hours, the zygote gives rise to an ookinete, a motile stage of the parasite life cycle that migrates out of the blood bolus and traverses the peritrophic matrix. The ookinete then penetrates the midgut epithelium at the apical junction of two epithelial cells and may transiently traverse several cells before exiting the basolateral membrane of the midgut epithelium. There it stops beneath the basal lamina (BL) and transforms *via *a took stage [5] into a sessile spherical oocyst [4].

The oocyst is the longest developmental stage of the *Plasmodium *life cycle, lasting between 10–15 days dependent upon the species [6]. During this time the oocyst will grow in size from ~5 μm to 50 μm and simultaneously undergo several rounds of nuclear division resulting in the production of up to 8000 haploid nuclei [7,8]. Sporozoites are formed by budding from the sporoblast(s) (for a review see [9]), formed by the retraction of the oocyst plasma membrane from the oocyst capsule [8-12]. In contrast to other apicomplexan parasites *Plasmodium *oocyst capsules do not appear to have an operculum for the release of sporozoites [10], instead, mature sporozoites egress from any point of the mature oocyst [13]. The oocyst capsule is a clearly distinct, electron dense, layer 0.1 -1 μm thick that encloses the oocyst and separates it from the adjacent mosquito tissue and BL [8,11,12]. Recently a *Plasmodium *protein specifically expressed in the capsule, PbCAP380, has been described [8]. Although the complete molecular composition of the capsule remains unknown, it has been proposed that it is derived from both parasite and mosquito proteins [10,14]; a hypothesis that has received some support as transgluaminase activity in oocysts suggests that mosquito proteins could be cross-linked into the capsule [15].

The BL is a complex meshwork of molecules. In mammals, the major components are laminin, collagen IV, entactin (nidogen), and perlecan [16]. The BL surrounds all epithelial sheets and is secreted by the epithelial cells that rest on it [17]. It has been implicated in diverse cellular functions including the ability to induce cell differentiation, to influence cell metabolism, organise proteins in adjacent plasma membranes and mediate cell migration [16,17].

Similarly, the composition of the insect BL includes laminin, collagen IV, entactin, and perlecan [16,18-20]. A high degree of conservation of some BL proteins such as laminin and collagen type IV exists between mammals and insects [16,21] but no fibronectin, thrombosbondin, von Willebrand factor, or elastin homologues have yet been identified in the model insect *Drosophila melanogaster *[16]. In contrast to other multicellular organisms, it has been proposed that the insect BL is secreted by haemocytes rather than epithelial cells [22-24]. Although the origin of insect BL is debatable [19,21,23,25,26], there is evidence for haemocyte involvement in BL formation and repair. For example, haemocytes associate with the BL during metamorphosis and wound repair [27] and mRNA for BL components such as collagen IV is expressed in haemocytes [28].

It has been suggested that interactions with the BL are crucial for *Plasmodium *development within its mosquito vector [10,25,29-33]. Several key observations that led to the proposal of a role for the BL in oocyst development include: the developmental site of the oocyst beneath the BL [33,34], the binding of parasite proteins to the BL [29,30,35,36] and the reduction in the intensity of oocysts infection when BL components, or parasite proteins that interact with them, are knocked-down or disrupted, respectively [25,29,37,38]. Furthermore, injection of ookinetes into the haemocoel of *D. melanogaster *led to the development, and eventual sporogony, of oocysts that were attached to the BL of various organs [39].

Adhesion of laminin to ookinetes has been demonstrated *in vitro *[35] and *in vivo *[25,40] and several proteins expressed in ookinetes have been shown to bind to laminin (discussed in [41]). Although it has now been demonstrated that an association with BL components is not a requirement for transformation of ookinetes to oocysts *in vitro *[5], recent evidence points towards a role in parasite evasion of the mosquito immune response [10,19,25,40]. In particular, the melanisation of Sephadex beads by *Aedes aegypti *mosquitoes is greatly reduced when they are coated with the surface protein of *P. gallinaceum *ookinetes, Pg28, to which laminin was found to be bound [40]. Ookinetes are 'covered' in laminin prior to the completion of their migration through the midgut epithelium [25] and *P. gallinaceum *oocysts have been labelled by collagen IV and laminin antibodies *in vivo*, leading to the suggestion that the capsule of *Plasmodium *oocysts is composed of both parasite- and mosquito-derived proteins [10]. It should be noted that light microscopy does not allow for the distinction of the capsule from the BL, which may explain why observations at the electron and light microscopy level have produced conflicting hypotheses concerning the relationship between the BL and oocysts. However, most authors agree that the BL is separate and morophologically distinct from the oocyst capsule [12,14,42,43]. In addition, some authors have proposed a more intimate association between the two, particularly with regards to the origin of the capsule [10,14]. For example, Melhorn *et al*. [14] described electron dense areas in the host cell adjacent to the oocyst capsule and surmised that material was secreted by the host-cell at these sites and used in the formation of the capsule. The role of the BL in oocyst development remains poorly understood, in particular with regards to the possible incorporation of BL components into the oocyst capsule.

Using post-embedded immunogold labelling electron microscopy, we show that the BL is in very close contact with, but distinct from, the capsule of *P. berghei *oocysts. In addition, we show that mosquito-derived laminin is incorporated into *P. berghei *oocysts 14 days post-infection. Finally, we present evidence that laminin may be produced by, or trafficked through, the midgut epithelium and discuss the implications of these results with regards to parasite development from the ookinete to the sporozoite.

## Results

### General observations

**Figure 1:**
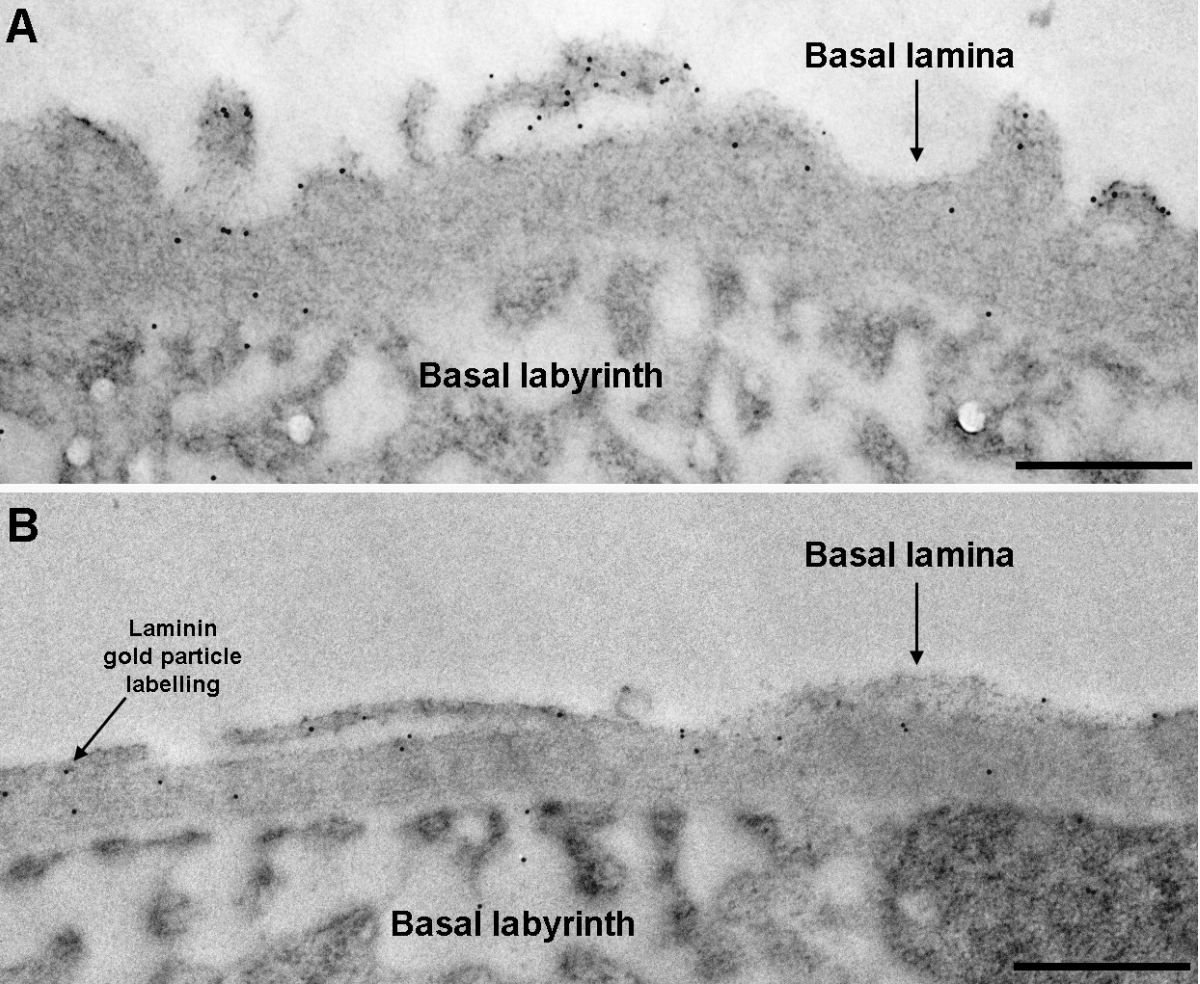
**Transmission electron micrographs of immunogold-labelled *P. textbfberghei*-infected and non-infected *An. stephensi *midguts demonstrating the presence of laminin in the basal lamina**. Basal lamina surrounding the midgut epithelium of *An. stephensi *mosquitoes (36,000 ×; scale bar = 500 nm). The distribution and density of laminin labelling (1:4000, 10 nm gold particles) is similar in both the A) uninfected and B) infected midguts.

The midgut epithelia of *An. stephensi *uninfected and infected mosquitoes were surrounded with a BL of similar thickness (between 100 – 500 nm) in all sections studied (Figure 1A and 1B). An infected midgut containing 32 oocysts and an uninfected midgut were selected for sectioning and subsequently, detailed analyses of laminin labelling were performed on five ultrathin sections from the infected midgut and four from the uninfected midgut.

Chain-specific β and γ *Drosophila *laminin antibodies [44] were used to label laminin in the ultrathin sections. These antibodies did not recognise mouse laminin, as determined by immunoblotting, but recognised a single band each when tested against the culture supernatant of *Drosophila *S2 cells (data not shown). In addition, labelling of whole *Anopheles *midguts was observed by indirect immunfluorescence assays (data not shown). No labelling was observed when a control rabbit IgG antibody (Sigma) was used, or when the primary antibody was omitted.

In order to further ensure that any labelling was not due to non-specific binding of the antibodies to the tissues, the density of gold particles in regions of interest was compared to control areas, namely; the resin in which the sample was embedded and the gut lumen. Labelling in these areas could indicate one of two different types of problems, non-specific binding resulting from lack of antibody specificity, or an excess of labelled antibody. The resin, which contained no biological material, was also used to verify the optimum concentration of antibodies to be used. The midgut lumen provided a biological control for the specificity of the antibodies, as laminin was not expected to be present there. Virtually no labelling was detected in the resin surrounding the sections at the antibody dilutions used in this study.

**Figure 2:**
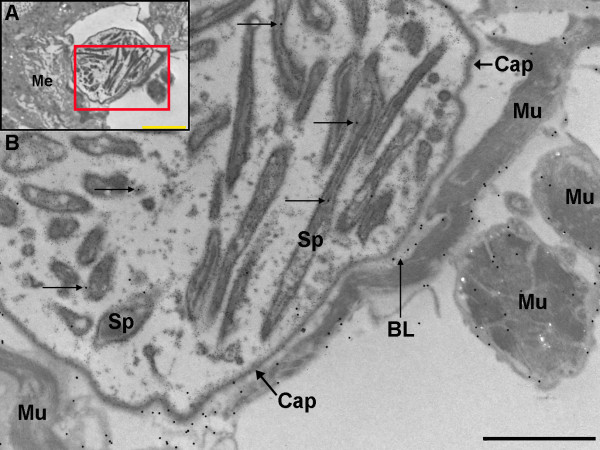
**Transmission electron micrograph of immunogold labelling for laminin in a *P. berghei *oocyst 14 days post-infection**. A) Oocyst underneath the basal lamina (5,000 ×; scale bar = 5 μm). B) Higher magnification image of boxed area of A (12,000 ×; scale bar = 2 μm). Laminin labelling (30 nm gold particles) is found close to the membranes of, or within, developing sporozoites (arrows). Laminin is also present on basal lamina around the midgut and individual muscles blocks. Me = midgut epithelium, BL = basal lamina, Sp = sporozoites, Cap = capsule, Mu = muscle.

**Figure 3:**
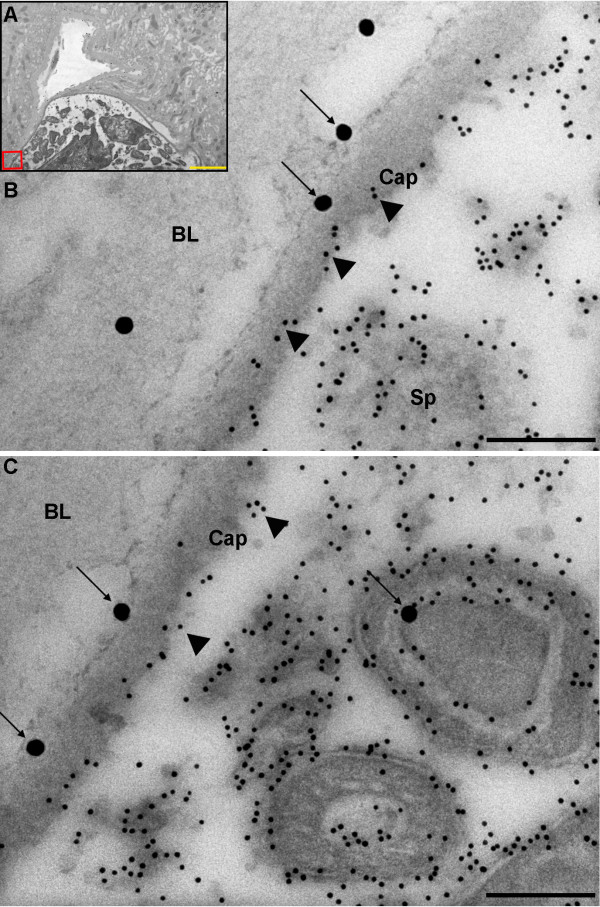
**Transmission electron micrograph showing the localisation of *An. stephensi *laminin and *P. berghei *circumsporozoite protein 14 days post-infection**. A) The inset shows the position of the oocyst in the midgut (10,000 ×; scale bar = 2 μm). B) Boxed area shown in A (120,000 ×; scale bar = 200 nm). C) Additional area of the same oocyst (120,000 ×; scale bar = 200 nm). Laminin (1:400) = 30 nm gold particles (arrows), CSP (1:1000) = 10 nm gold particles (arrowheads). Note the presence of labelling for laminin within the sporozoite and on the outer surface of the capsule. BL = basal lamina, Sp = sporozoites, Cap = capsule.

**Figure 4:**
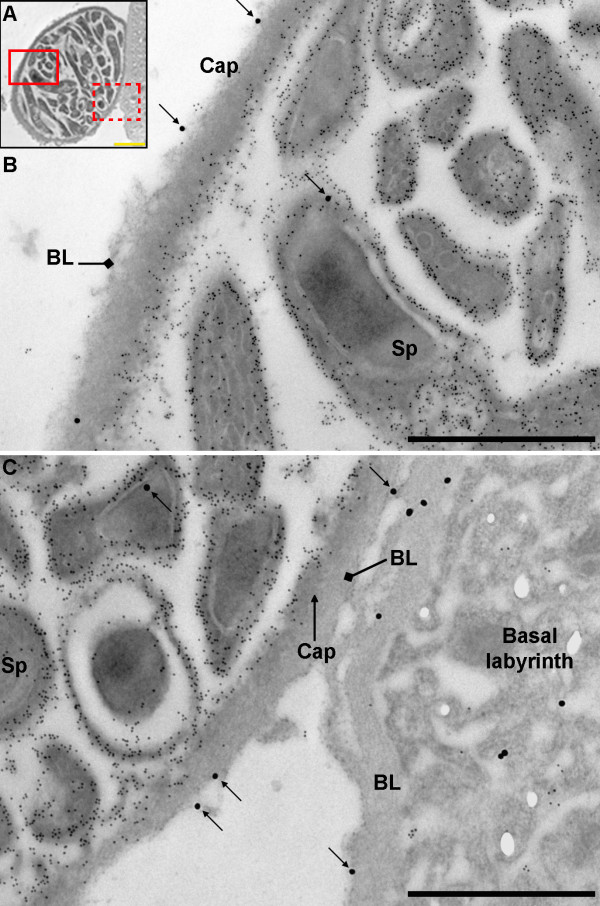
**Transmission electron micrograph showing laminin and circumsporozoite protein distribution in *P. berghei *oocysts 14 days post-infection**. A) Oocyst on the edge of the midgut epithelium (12,000 ×; scale bar = 2 μm). B boxed area with solid line, C) boxed area with dotted line (40,000 ×; scale bar = 1 μm). The oocyst capsule appears darker than the layer of basal lamina coating it. Remnants of basal lamina are seen on the edge of the oocyst facing the haemocoel (B) and a thicker layer is seen adjacent to the basal labyrinth of the midgut epithelium (C) (diamond arrows). The majority of 30 nm gold particles indicating laminin labelling (1:800) (arrows) are found on the outer surface of the oocyst capsule. Note that some laminin labelling is also found in association with developing sporozoites The 10 nm gold particles correspond to circumsporozoite protein labelling (1:4000). BL = basal lamina, Cap = capsule, Sp = sporozoite.

Oocysts at different stages of development, the majority of which contained fully formed sporozoites, were observed 14 days post-infection. A capsule was plainly visible surrounding the oocysts and clearly distinct from the BL. Laminin labelling using 30 nm gold particles was observed around the oocyst capsules and within the oocysts; primarily associated with developing sporozoites (Figure 2B). As no capsule-specific antibodies were available at the time, circumsporozoite protein (CSP) was used as a marker to delineate oocyst-specific structures, as it is not located within the capsule. Some infected midgut sections were also labelled with CSP using 10 nm gold particles and labelling was subsequently observed within *P. berghei *oocysts, around developing sporozoites and adjacent to the inner surface of the capsule (e.g. Figure 3B, 3C, 4B and 4C) as reported by previous studies [45-48]. Inspection of the sections revealed that a layer of BL of variable thickness surrounded oocysts in very close association with the oocysts' capsule. This layer of BL appeared to be thinner in areas of oocysts that would have protruded into the haemocoel than in areas of the capsule that were adjacent to the basal labyrinth of the midgut epithelial cells (Figure 4C).

### Laminin distribution in *An. stephensi *midgut epithelial cells

Laminin was initially labelled using 30 nm gold particles and similar distribution patterns in the BL of uninfected and infected mosquito midguts were observed (Figure 1A and 1B). Particle size was then reduced to 10 nm as these gave a greater accuracy of labelling and a comparison of the density of laminin labelling associated with the midgut BL and control areas (resin and midgut lumen) demonstrated that BLs from both the infected and uninfected midguts contained a significantly higher density of gold particles than the control areas (Kruskal-Wallis: H_(7) _= 59.8, *P *< 0.001; Table 1). As can be seen from data in Table 1, some labelling was detected in the midgut lumens and this was at a higher density in the infected midgut sections compared to the uninfected sections. The reason for this observation is unclear; however, in both cases, laminin labelling of the midgut lumen was not significantly different from labelling in the resin. Importantly, no significant differences were detected in the density of labelling of the BL between the infected and uninfected midguts (Dunn's Test: Q_(7) _= 0.05, *P *> 0.5).

**Table 1:** Density of laminin labelling in the capsule of *P. berghei *oocysts.

	**Infected Midgut** (mean density/μm^2^)	**Uninfected Midgut** (mean density/μm^2^)
***P. berghei *oocyst capsule**	7.91 (± 0.82)^a^	N/A
*P. berghei *oocyst minus capsule*	1.35 (± 0.11)^b^	N/A
**Basal lamina**	14.00 (± 1.36)^a^	13.98 (± 1.71)^x^
**Midgut Epithelium^≠^**	1.02 (± 0.39)^b^	2.03 (± 0.80)^x^
**Midgut lumen**	0.89 (± 0.19)^b^	0.18 (± 0.05)^y^
**Resin**	0.30 (± 0.07)^b^	0.17 (± 0.04)^y^

There was significantly more labelling of laminin in the oocyst capsule and the midgut basal lamina than the control areas or *P. berghei *oocysts minus capsule. Values in brackets = standard error of the mean. Infected midgut: ^a ^indicates a significant difference from control areas denoted by ^b^. Uninfected midgut: ^x ^indicates a significant difference from control area denoted by ^y^. *Laminin distribution was analysed within whole oocysts (minus capsule) without any distinction made between sporozoites, cytoplasm or subcapsular space. ^≠ ^Analysis was performed on whole cells with no distinction between cytoplasm and organelles. In later analyses, laminin distribution in various organelles was compared to distribution in the midgut lumen.

**Figure 5:**
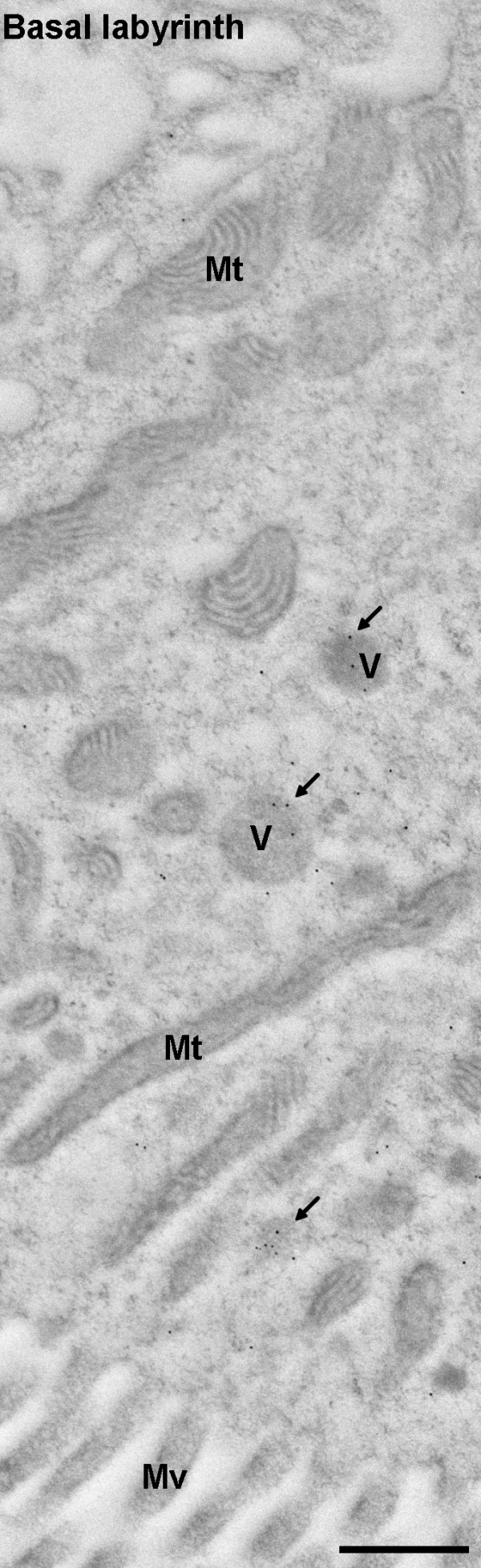
**Laminin distribution in midgut epithelial cells**. A section spanning the midgut epithelium from the basal labyrinth to the microvilli. Clusters of laminin labelling (1:4000) can be seen in vesicles (arrows) (36,000 ×; scale bar = 500 nm). Mv = microvilli, Mt = mitochondria, V = vesicles.

Laminin was found to be present within the midgut epithelial cells (Figure 5). The density of laminin labelling in the uninfected midgut cells was significantly higher than in the resin (Q_(3) _= 2.73, *P *< 0.05), and was not significantly different from the density of labelling in the BL (Q_(3) _= 2.15, *P *> 0.2). In midgut epithelium from infected mosquitoes the density of labelling was significantly higher in the BL than in the resin, (Q_(3) _= 5.03, *P *< 0.001), however, there was no significant difference between labelling in the midgut epithelium and resin (Q_(3) _= 1.36, *P *> 0.05).

Gold particles were observed in clusters, particularly in the apical region of the cells, adjacent to the midgut lumen. This area contains vesicles, secretory organelles such as the endoplasmic reticulum and the Golgi complex, as well as mitochondria [49,50]. Gold particle clusters appeared to be associated with vesicles (Figure 5) leading to a more detailed analysis of laminin distribution within the midgut epithelial cells (see Methods). In some sections parts of the secretory machinery could be distinguished, however, as LR white resin embedding does not allow for good preservation of membranes, localisation of gold particles within the ER or other organelles could not be reliably ascertained. Thus, the density of gold particle labelling in vesicles, mitochondria, cytoplasm, microvilli, and midgut lumen was calculated. Significantly more gold particles were associated with vesicles than any of the other structures for the infected midgut cells (ANOVA: F_(4) _= 16.30, *P *< 0.001). Labelling of the vesicles in uninfected midgut cells was significantly higher than for any other cellular profiles (ANOVA: F_(4) _= 25.79, *P *< 0.001). Finally, there was a significant effect of infection, with laminin labelling higher in midgut cells from the infected mosquito sections than those from the uninfected ones (GLM: F_(4,1) _= 6.08, *P *= 0.016).

### Laminin incorporation into the oocyst capsule

**Figure 6:**
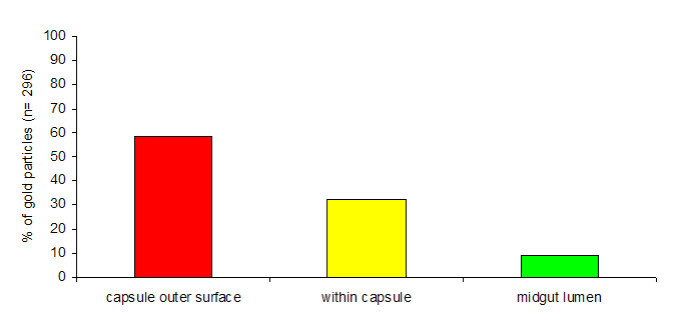
**Distribution of laminin in *P. berghei *oocyst capsules**. Ninety three areas of capsule from 11 *P. berghei *oocysts were analysed to determine the precise location of laminin by immunogold labelling using anti-*D. melanogaster *laminin antibodies directed against the β and γ chains and detected with a 10 nm gold-conjugated secondary antibody. The percentage distribution of gold particles (n = 296) situated either on the outer surface of the capsule, within the capsule or and equivalent are of midgut lumen was calculated.

**Figure 7:**
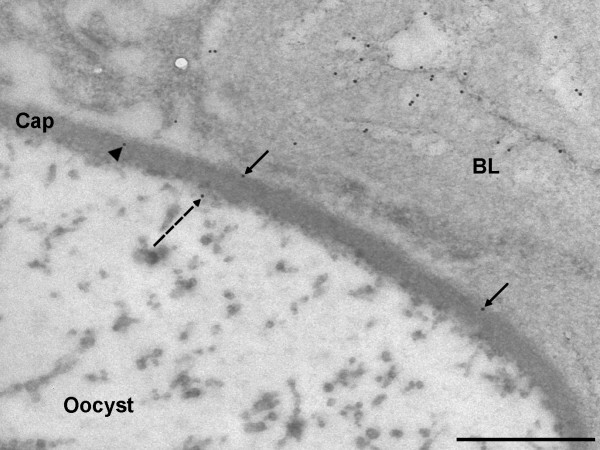
**Localisation of laminin in the capsule of *P. berghei *oocysts**. Sections of *P. berghei*- infected *An. stephensi *midguts were probed with anti- *D. melanogaster *laminin-1 antibodies (1:4000) followed by 10 nm gold particle-conjugated secondary antibodies. Laminin is present on the outer surface of the capsule (arrows), within the capsule (arrowhead), and on the inner surface of the capsule (dashed arrow) (60,000 ×; scale bar = 500 nm). Cap = capsule, BL = basal lamina.

Eleven oocysts were examined in the infected midgut sections and, where possible, 10 images were collected using a stratified random sampling method. Ninety three images were obtained, from which an analysis of the distribution of gold particles within the oocyst capsules was performed to determine whether the labelling observed reflected an incorporation of laminin into the capsule. The density of laminin labelling in the oocyst capsule was only 2-fold lower than in the BL of both infected and non-infected midgut sections (Table 1) and significantly more labelling was observed in or around the capsule than in the resin (Q_(3) _= 4.33, *P *< 0.001) and gut lumen (*P *< 0.05). Only sixty percent of the gold particles associated with the oocyst capsule were localised on the exterior surface of the capsule (Figure 6), indicating that, although laminin was primarily bound to the outer surface of the oocyst capsule, a considerable amount had been incorporated into the capsule (Figure 7). To confirm this, a comparison was made with an equivalent area of midgut lumen. The density of gold particles within the oocyst capsule was significantly higher than in the midgut lumen (Chi-square: χ^2^_(1) _48.6, *P *< 0.001) suggesting that laminin is indeed incorporated into the capsule.

### Laminin labelling associated with sporozoites

**Figure 8:**
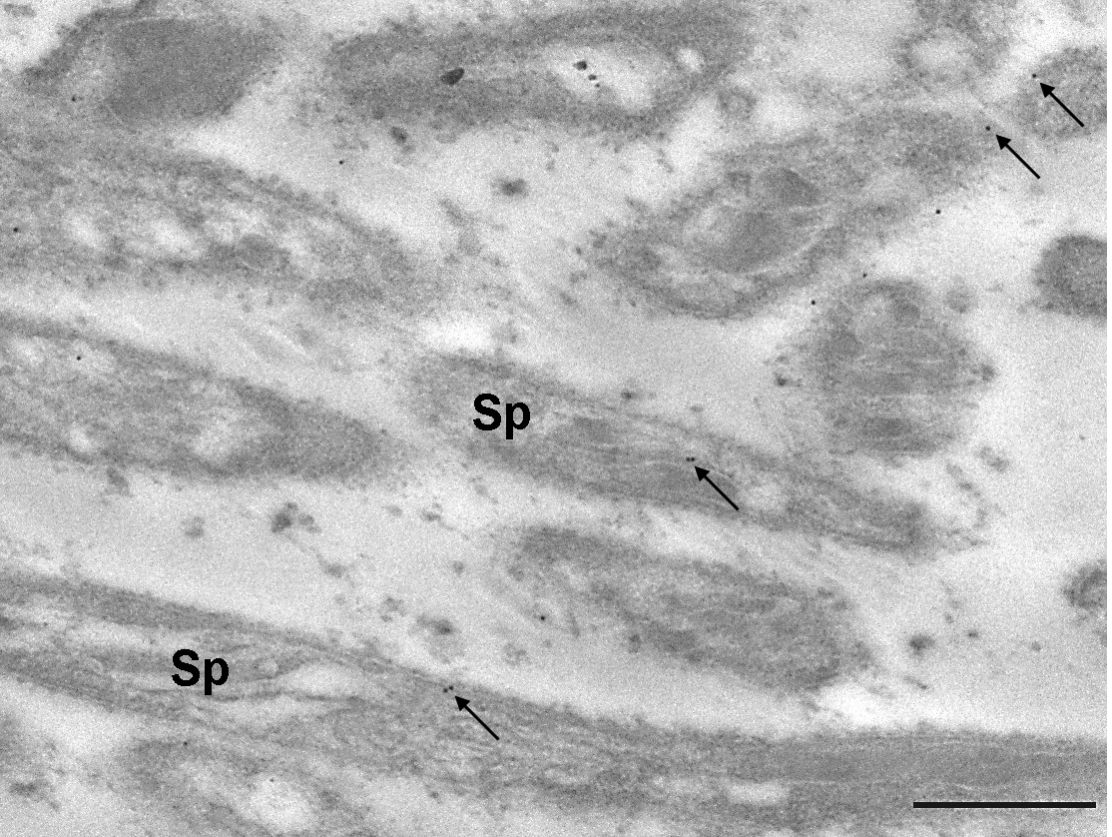
**Localisation of laminin in a *P. berghei *oocyst**. Sections of *P. berghei*- infected *An. stephensi *midguts were probed with anti- *D. melanogaster *laminin-1 antibodies (1:4000). Labelling was often observed within or near developing sporozoites (arrows) (36,000 ×; scale bars = 500 nm). Sp = sporozoites.

Even though labelling of laminin within oocysts as a whole was not significantly different from control areas (Table 1, oocysts minus capsule) an examination of the sections suggested a systematic association of laminin gold particle-labelling with sporozoites (Figure 8). In order to assess whether this labelling pattern was real, or a visual artefact due to non-specific binding, gold particle distribution was analysed so that a distinction was made between gold particles associated with developing sporozoites and those in the oocysts' cytoplasm again using 10 nm particles, (see Methods for further details). A significantly higher proportion of gold particles was found to be localised in developing sporozoites than in the oocyst cytoplasm (*t*-test: *t*_(9) _= 3.54, *P *= 0.003) indicating a specific association of laminin with sporozoites within oocysts. These observations may reflect an actual binding of the antibodies to laminin located within sporozoites, or cross-reactivity with sporozoite protein(s). The former would imply that laminin is internalised into the oocyst and then binds to sporozoite molecules, the latter, that one or more sporozoite proteins contain laminin-like domains. Cross reactivity with circumsporozoite protein (CSP) was considered as this protein has been reported to be localised on the inner surface of the capsule and on sporozoites, and indeed our observations confirm this. However, the distribution of laminin was not consistent with the localisation of CSP on the inner surface of the capsule.

To determine if cross-reactivity may have occurred with any sporozoite stage proteins, a sequence similarity search against the *Plasmodium *genome was performed using *D. melanogaster *laminin sequences. The annotated sequences with the highest homology were the recently characterised cysteine repeat modular proteins (e.g. PbCRMP2, E-value = 4.0e-06, 36% similarity). They are predicted to be expressed on the parasite surface and to contain a single EGF-like domain proximal to a transmembrane domain [51]. Four distinct proteins have been characterised in *P. falciparum*, all of which are expressed in sporozoites (salivary gland: PfCRMP1 and 2; oocyst sporozoites: PfCRMP3 and 4) [51]. PfCRMP2, in particular, is expressed in sporozoites emerging from oocysts [51]. In addition, Thompson *et al*. [51] determined the expression of the *P. berghei *homologues and found that PbCRMP1 is expressed in young oocysts and sporulating oocysts, whereas PbCRMP2 is expressed in sporulating oocysts and sporozoites.

## Discussion

This study has clearly demonstrated that laminin, a major component of the midgut BL, coats the external surface of the oocyst capsule, which is clearly distinct from the BL, and is also incorporated into the capsule. Although the incorporation of other BL components into the capsule was not investigated here, it is conceivable that collagen IV and other molecules could also form a coating over the oocyst capsule, adding to the disguise [10].

The presence of laminin labelling within oocysts, and specifically associated with sporozoites, was unexpected. We believe it was synthesised by the mosquito, not the parasite. The oocyst capsule is permeable to amino acids (Vanderberg and Rozeboom, as cited in [12]) and it is probable that macromolecules such as laminin also pass through it. The mechanism by which laminin would then be internalised into the oocyst, which would require it to pass through the oocyst plasma membrane, is unclear. The parasitic protozoans *Tritrichomonas foetus *and *Trichomonas vaginalis *have been reported to ingest latex or micron beads coated with laminin by pinocytosis [52,53]. It is possible that *Plasmodium *could have a similar mechanism for laminin uptake.

Since single cell organisms are not believed to be able to synthesise BLs or the complex proteins that constitute them [54] we must also consider whether the anti-laminin antibodies used were binding non-specifically to other proteins. The major ookinete surface proteins of *Plasmodium*, P25 and P28, contain EGF-like repeats [55,56] which are characteristic of laminins. However, labelling of the ookinete surface was not observed with the antibodies used in this study (data not shown), a finding also reported by Arrighi *et al*. [25] using anti-mouse-laminin antibodies. Therefore if this motif were recognised, it would not, in itself, be sufficient for labelling by these antibodies. This suggests that cross-reactivity with these proteins does not occur. With regards to other proteins expressed in the oocysts, the cysteine rich modular proteins (PxCRMP) were also considered as candidates for cross-reactivity, however, we think this is unlikely as these proteins only contain a single EGF-like domain, unlike P28, P25, and laminin [51]. It remains to be confirmed whether the gold labelling was indeed specific for laminin or whether anti-laminin antibodies are also binding to *Plasmodium *proteins.

Within the midgut it has been shown that laminin is upregulated after a blood meal and the BL itself thickens post-blood feeding [19,25,57]. A number of morphological changes rapidly occur within the midgut epithelium following a blood meal, including the accumulation of mitochondria and secretory vesicles in proximity to the brush border, the unfolding of the basal labyrinth (thus increasing surface area) and an increase in protein abundance [50,58]. In addition, it has been reported that collagen IV expression shows a 2-fold increase during oocyst development when compared to expression levels following an uninfected blood feed. Gare *et al*. [19] speculated that the upregulation of collagen IV and possibly other BL proteins during infections are akin to a wound healing response in infected mosquitoes. However, it has been reported that fourteen days post-blood feeding (the time of this study) the midgut epithelium and BL have recovered from the stresses induced by the presence of a blood meal [19,50,58]. Furthermore, the development of a new BL around the midgut epithelium ceases between 10–15 days post-infection when repair has been achieved [19]. In this study, the thickness of the BL and the density of laminin therein did not differ between infected and uninfected *An. stephensi *fourteen days post-blood feeding.

Laminin was detected within the midgut epithelial cells. It was concentrated in vesicles that were mostly located at the apical end of the cells, in areas containing large numbers of mitochondria. Laminin labelling in the uninfected *An. stephensi *midgut epithelial cells was significantly higher than in the resin or midgut lumen, suggesting that laminin may be synthesised by midgut epithelial cells and exported *via *vesicles to the BL. Although our observations of laminin distribution in the midgut epithelium are based on sections from single infected and uninfected midguts and must therefore be interpreted with caution, studies examining the expression of BL components have shown that both collagen IV and laminin are expressed in the midgut epithelium [19,20,25]; lending support to our findings. Endocytosis of laminin has been reported to occur in mammals (a process associated with the turnover of the extracellular matrix during wound healing and growth [59]); a similar process could also be occurring here.

The BL has been shown to be an important indicator of 'self' in insects [60]. Parasites able to bind BL components to their surface could appear as 'self' and thus evade recognition by the mosquito's immune system [10,20,29,30,35,36]. For example, recombinant *P. gallinaceum *P28, mouse laminin, and *Drosophila*-conditioned cell culture medium (known to contain BL components) all contribute to a reduction in the melanisation of Sephadex beads by *Ae. aegypti *[40]. Furthermore, *P. berghei *ookinetes are coated by laminin prior to the completion of their passage through the midgut of *An. gambiae *[25]. It is likely that this coating will assist with evasion of the immune system. Adini and Warburg [10] proposed that mosquito BL proteins were incorporated into the capsule of *Plasmodium *oocysts and speculated that this would enable them to be masked from the immune response of the vector. Oocysts produced *in vitro *typically do not form a distinct capsule ([31,32,61] (personal observations), suggesting an essential role for mosquito-derived proteins in the formation of this structure. Furthermore, it has recently been shown that PbCAP380-disrupted parasites, that are not able to form a capsule, do not survive in the mosquito midgut [8]. The authors speculated that this protein could be implicated in the incorporation of mosquito molecules into the capsule; a key interaction that, when interrupted, results in parasites that are unable to mask themselves from the mosquito's immune system [8].

At 14 days post-infection, most of the oocysts in this study were not surrounded by a thick and continuous BL on the haemocoel side. Currently, the widely accepted model to explain the observation that oocysts are coated in BL components is that the extracellular matrix is secreted by haemocytes [62]. If so, then this secretion could be related to their function as immune competent cells as it has been reported that the melanotic encapsulation of pathogens is terminated by the addition of basement membrane-like layer [63]. However, one must question why, if haemocytes are responsible for producing the midgut BL, new BL is not continuously being laid down as the oocysts grow and expand. If the formation of the BL is essentially dependent upon synthesis by midgut epithelial cells, one would expect the BL to be present as a thicker layer on the parts of the oocyst adjacent to the basal labyrinth, as was frequently observed in this study and has previously been reported [19]. The observations presented here, and those of Arrighi *et al*. [25] and Gare *et al*. [19] demonstrating that laminin and collagen IV, respectively, are transcribed in the midgut epithelium, strongly suggest a role for midgut epithelial cells in the production of the BL. These two potential sources of BL need not, however, be mutually exclusive. BLs have important biological functions and it is possible that the midgut epithelium and haemocytes both contribute to their synthesis.

**Figure 9:**
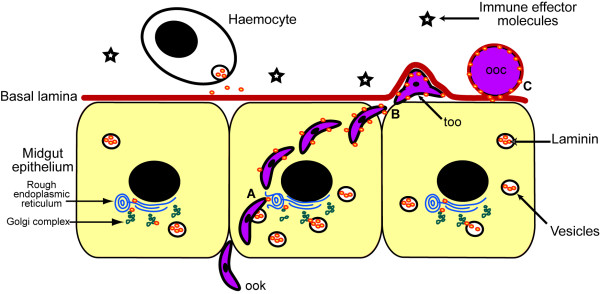
**Proposed model of interaction between *P. berghei *sporogonic stages and *An. stephensi *laminin**. A) Ookinetes (ook) are coated with laminin produced by midgut epithelial cells when they migrate through these cells as they disrupt membranes of the secretory machinery during migration. B) This enables them to mask themselves from the immune system when they reach the basal lamina and begin transformation *via *a took stage (too). C) The basal lamina is stretched by the developing oocysts (ooc) and laminin is replaced beneath the oocysts by production in the midgut cells. Haemocytes may also contribute to the midgut BL.

Our observations have led us to propose the following model (outlined in Figure 9) for further investigation. Laminin, synthesised by midgut epithelial cells, adheres to and coats ookinetes during their passage towards the BL, as proposed for β integrin coating [64]. Upon contact with the BL, ookinetes stop migrating, perhaps because of an inhibitory role of laminin on locomotion, or perhaps because ookinetes bind to the BL and can no longer glide. Transformation is initiated here and nutrients circulating in the haemolymph are probably required for the complete transformation of ookinetes into oocysts [5]. As oocysts grow, laminin and perhaps other BL components continue to be secreted by the epithelial cells and both coat and are incorporated into the oocyst capsule. Whether the observed incorporation of laminin into the oocyst capsule is reflective of laminin forming an integral part of the capsule, or just passing though it, remains to be elucidated.

## Conclusion

Here we show by semi-quantitative immunogold labelling that, at 14 days post- infection, laminin coats and is incorporated into the capsule of *P. berghei *oocysts. Immunogold labelling for laminin was also observed in sporozoites within maturing oocysts and we suggest a putative role for laminin in protecting both the oocyst and sporozoite from the mosquito defence system. In addition, we show that laminin is associated with vesicles present in the midgut epithelial cells. This observation has led us to propose that ookinetes traversing the midgut may become coated in laminin, and possibly other BL components that would enable ookinetes to evade the vector's immune system as they emerge from the epithelial cells.

## Methods

### Mosquito infections

Adult female *An. stephensi *Liston (Dubai Strain) mosquitoes were allowed to blood feed on a male CD mouse infected with *P. berghei *ANKA_CON _[65] for 35 min. A control blood feed on an uninfected CD male mouse was performed in parallel with mosquitoes from the same generation. After blood feeding, mosquitoes were transferred to a 19°C incubator and maintained on a 4% glucose solution containing 0.05% *para*-aminobenzoic acid, 200 U/L penicillin, and 200 μg/L streptomycin. Unfed mosquitoes were removed from each treatment cage 24 h post-blood feeding. Experiments were performed using approved protocols in accordance with the UK Animals (Scientific Procedures) Act 1986 under licence from the UK Home Office.

### Fixation and embedding

*P. berghei *GFP_CON_-infected *An. stephensi *midguts were monitored 14 days post-infection by fluorescence microscopy. Three infected and five uninfected midguts were fixed in 4% paraformaldehyde containing 0.1% glutaraldehyde in 0.1 M phosphate buffer (PB) for 2 h on a rotator. The samples were washed three times for 5 min in 0.1 M PB and dehydrated in a graded ethanolic series. The midguts were then infiltrated with LR-White Hard Grade Acrylic Resin (Agar Scientific) for 2 h on a rotator. Samples were infiltrated twice more with fresh LR-white resin, and a further infiltration step was performed overnight. Following an additional 2 resin changes, samples were transferred to capped gelatine beam capsules and polymerised for 24 h at 50°C. Using a Leica Ultracut UCT Ultramicrotome 100 nm ultrathin sections were taken transversally through an infected and uninfected midgut and collected onto 200 mesh thin bar nickel grids (Product code: G2002N, Agar Scientific).

### Antibodies and specificity controls

Laminin was detected in *An. stephensi *midgut sections using affinity purified rabbit polyclonal antibodies raised against the β and γ chains, respectively, of laminin secreted by *D. melanogaster *Kc167 cell cultures [44] at 1:800 or 1:4000 dilutions. Some sections were also labelled with a *P. berghei *circumsporozoite protein (CSP) mouse monoclonal antibody (3D11) at 1:100 or 1:4000 depending on the secondary antibody gold particle diameter. Labelling controls included a rabbit IgG antibody (Sigma) or omission of the primary antibody.

### Post-embedded immunogold labelling

Labelling was performed in a humid chamber by immersing grids in a series of reagents. Grids labelled for the quantitative analysis were immersed in the same drop of each reagent to ensure that samples (infected and uninfected midguts) were labelled in the same conditions. Prior to labelling, grids were placed in 40 μl of 0.05 M Tris buffered saline, (TBS) pH7.4, for 5 min and blocked for 30 min in 20% goat serum (Harlan Sera Lab) in TBS. Grids were then incubated overnight at 4°C in a mixture of both primary antibodies (CSP 1:1000 or 1:4000; laminin 1:400 or 1:800) diluted in 1% goat serum in TBS (GS-TBS). Following three ten minute immersions in TBS, samples were blocked for 15 min with 20% GS-TBS. Grids were immersed for 1 h at room temperature with anti-mouse IgG-10 nm gold-conjugated antibody (British Biocell) mixed with anti-rabbit IgG-30 nm gold-conjugated antibody, each diluted 1:20 in 1% GS-TBS. Semi-quantitative analyses were performed by labelling only with laminin (1:4000) followed by an anti-rabbit IgG-10 nm gold-conjugated antibody. Grids were stained with 2% aqueous uranyl acetate for 20 min and dried prior to examination with a JEOL 1230 transmission electron microscope (TEM) and/or a JEOL 100 CXII TEM. Images were acquired with a MegaView III digital camera (Soft Imaging Systems) using iTEM software analysis (Soft Imaging Systems). Several sections (3–5) were observed for all experiments.

### Semi-quantitative analysis of laminin labelling of *P. berghei *oocysts and *An. stephensi *midguts

The density of laminin labelling (gold particles/μm^2^) was determined in portions of oocyst capsule, oocysts, the BL, midgut epithelium, gut lumen, and resin for both the infected and uninfected midguts. Images were acquired at a fixed magnification of either 60,000 × (JEOL 1230) or 36,000 and 58,000 × (JEOL 100 CXII).

Areas of oocyst capsule used for the analysis were selected using a stratified random sampling method. Briefly, the selection of oocysts was not random (all were sampled) but for each oocyst the initial area chosen for counts was selected randomly by reducing the magnification to a level where the gold particles were indiscernible, images were then acquired for every other field of view around the oocyst and 93 images were acquired in total. For all other areas of interest, namely: oocysts, BL, midgut epithelium, gut lumen, and resin, areas were selected at random and 10 images were acquired per region of interest.

To investigate if an association existed between laminin labelling and developing sporozoites, which appeared to be more densely labelled than oocyst cytoplasm, the data were analysed following a method described by Mahendrasingam and colleagues [66]. Briefly, an overlay containing regularly spaced lines was placed onto the images using the GNU Image Manipulation Software . The number of points touching and/or lying within sporozoites and cytoplasm was counted to estimate the proportion of oocyst occupied by each of these regions, and the number of gold particles falling into each region was counted. Gold particles were only deemed to be associated with sporozoites if they were located within them or on the membrane. When gold particles were located within 1 particle diameter of each other, a count of only 1 was given, as it is likely that those particles were binding to the same site. The total area of oocyst for each image was measured using iTEM analySIS software (Soft Imaging Systems) and the proportion of the area occupied by sporozoites and cytoplasm was determined. Using these data, the density of gold labelling (number of gold particles/μm^2^) was calculated.

In order to investigate whether laminin present in midgut epithelial cells, was associated with any cellular structures (e.g. vesicles) 10 images of midgut epithelium were acquired for each midgut (infected and uninfected). Preliminary observations suggested a denser pattern of labelling on the lumenal side of the midgut, thus images were acquired at random within this region and analysed as described above [66]. The density of immunogold labelling within vesicles, mitochondria, microvilli, gut lumen and cytoplasm was calculated.

### Laminin-like sequences in *Plasmodium*

To determine whether cross-reactivity could have occurred with proteins from any stage of *Plasmodium*, sequences for the *Drosophila *laminin α1 [GenBank: AAA28662], β [GenBank: AAD19752], and γ [GenBank: AAA28664] chains were retrieved from the National Center for Biotechnology Information (National Library of Medicine and National Institutes of Health) protein sequence database using Geneious Pro version 2.5.4 (Biomatters Ltd, [67]). These sequences were used to search for laminin-like sequences by using the NCBI Basic Local Alignment Search Tool (BLAST) against the apicomplexan database.

### Statistical analysis

A comparison of the density of laminin labelling in the oocyst capsule and BL with control areas such as resin and gut lumen was analysed with the non-parametric Kruskal-Wallis test in conjunction with Dunn's test for multiple comparisons [68]. The distribution of gold particles within the capsule, indicative of incorporation, was analysed with a Chi-square goodness of fit test. The density of gold particles in association with sporozoites was analysed using a one-tailed 2-sample *t*-test. Finally, the association of laminin with cellular structures within infected and uninfected midgut epithelial cells was analysed on transformed data with a one-way ANOVA and a General Linear Model [68]. Statistical analyses were performed with Minitab^® ^15 (ANOVA, GLM, and Chi-squared) and GraphPad Prism^® ^version 5 (Kruskal-Wallis and Dunn's Test).

## Competing interests

The authors declare that they have no competing interests.

## Authors' contributions

AN performed the mosquito infections and immunogold electron microscopy labelling. KW sectioned the midguts and aided in the acquisition of data. HH conceived of the project and AN and HH prepared the manuscript.
